# Sulfur-Containing Amino Acids: The Conversion Process from Product to Substrate

**DOI:** 10.3390/ijms27114771

**Published:** 2026-05-26

**Authors:** Peining Zheng, Yuanting Jin, Chong Wei, Chaoyi Xia

**Affiliations:** 1 College of Life Sciences, China Jiliang University, Hangzhou 310018, China; p24091055086@cjlu.edu.cn (P.Z.); jinyuanting@cjlu.edu.cn (Y.J.); 2 Zhejiang Provincial Engineering Research Center of New Technologies and Applications for Targeted Therapy of Major Diseases, Zhejiang Provincial Key Laboratory of Drug Discovery and Development for Metabolic Diseases, College of Life Sciences and Medicine, Zhejiang Sci-Tech University, Hangzhou 310018, China; 2024010901005@mails.zstu.edu.cn

**Keywords:** cysteine, methionine, synthetic metabolism

## Abstract

Methionine and cysteine are the principal sulfur-containing proteinogenic amino acids, playing pivotal roles in protein structure, function, and cellular metabolic regulation. The biosynthetic machinery of these amino acids is intricate and exhibits distinct evolutionary divergence across species. This review comprehensively summarizes the biosynthesis of cysteine and methionine in model organisms ranging from *Escherichia coli* and yeast to plants and *Homo sapiens*. Specifically, we examine the metabolic interconversion and the transition of roles between these two amino acids during de novo synthesis. Furthermore, we dissect the physiological significance of the transsulfuration pathway in mammals, which utilizes methionine as a precursor for cysteine biosynthesis.

## 1. Introduction

Among the classical amino acids that constitute proteins, cysteine and methionine hold a unique status as the sole sulfur-containing members, acting as critical sinks for reactive species due to their specific atomic composition [1]. The remarkable biological versatility of cysteine stems directly from the chemical properties of its thiol group, which is highly nucleophilic and redox-active, allowing it to function as an effective molecular switch in response to cellular stimuli [2]. This reactivity is fundamental to its direct participation in the active sites of numerous enzymes, such as cysteine cathepsins, where it performs vital catalytic functions ranging from protein turnover to antigen presentation [3]. Furthermore, the ability of cysteine residues to form covalent disulfide bonds is crucial for maintaining the structural integrity of proteins, a feature that can be mapped with high precision to understand protein stability [4]. Cysteine is also a primary target for a host of post-translational modifications, including *S*-glutathionylation, a key mechanism for regulating protein function and achieving antioxidant defense through redox signaling [5]. Beyond its role in protein architecture, cysteine serves as the rate-limiting precursor for the synthesis of glutathione (GSH), and its availability is tightly linked to cell death pathways like ferroptosis when regulation fails [6].

Concurrently, methionine fulfills two equally indispensable roles, primarily serving as the conserved initiating amino acid for the synthesis of nearly all eukaryotic proteins, mediated by specific quality control mechanisms [7]. In the realm of cellular metabolism, its more profound function is as the exclusive precursor to *S*-adenosylmethionine (SAM), a vital metabolite that links nutritional status to autophagy and cell growth [8]. SAM acts as the universal methyl donor, participating in the enzymatic methylation of a wide array of biological substrates, including DNA and histones, thereby playing a central role in the epigenetic regulation of gene expression [9]. Moreover, SAM is a critical metabolic node, providing aminopropyl groups for polyamine synthesis and acting as an allosteric regulator to coordinate sulfur flux through the transsulfuration pathway [10].

The profound biological importance of cysteine and methionine necessitates robust mechanisms in all organisms to ensure their continuous and regulated supply, often integrating these pathways with carbon and nitrogen metabolism [11]. However, the strategies employed to achieve this metabolic homeostasis reveal a fundamental evolutionary divergence, particularly regarding assimilatory sulfate reduction pathways [12]. Mammals exhibit a pronounced metabolic dependency. Because they lack the capacity for strictly de novo methionine biosynthesis, it is classified as a nutritionally essential amino acid that must be primarily obtained from the diet to maintain post-translational regulatory networks [13].

In mammals, cysteine is considered conditionally essential. This classification arises because, although endogenous synthesis is generally sufficient for healthy adults, it becomes critically inadequate under specific physiological or pathological conditions, such as rapid neonatal growth, severe metabolic stress, or critical illness. In such scenarios, the organism’s metabolic demand significantly outpaces its biosynthetic capacity, making adequate dietary supplementation of cysteine strictly indispensable [14]. Under normal conditions, however, adequate cysteine is maintained via diet or the irreversible transsulfuration pathway, which concurrently generates hydrogen sulfide. This pathway channels the sulfur atom from dietary methionine through the intermediate homocysteine into a serine backbone to generate cysteine. The process is governed by complex enzymatic regulators, most notably cystathionine β-synthase [15].

In stark contrast, plants and many microorganisms display significant metabolic autonomy. They generally retain complex and energetically costly de novo biosynthetic pathways, allowing them to synthesize both cysteine and methionine from simple inorganic sulfur sources [16]. This metabolic dichotomy separating organisms capable of primary sulfur assimilation from those dependent on dietary transsulfuration, represents a key evolutionary divide with far-reaching implications for nutrition, toxicology, and disease pathology.

In this review, we first conduct a comparative analysis of the anabolic processes of cysteine and methionine across diverse model organisms, ranging from *E. coli* and yeast to plants and humans. Subsequently, we explore the evolutionary plasticity that has shaped these diverse metabolic networks and the intricate mechanisms regulating their function.

## 2. The Synthesis of Cysteine and Methionine in *Escherichia coli*

**Figure 1:**
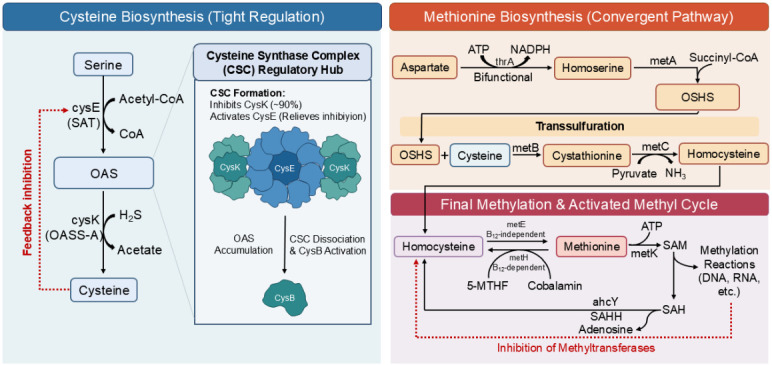
*Escherichia coli* Cysteine and Methionine Biosynthesis. Abbreviations: Acetyl-CoA, Acetyl-coenzyme A; SAT, Serine *O*-acetyltransferase; OAS, *O*-acetylserine; OASS-A, *O*-acetylserine sulfhydrylase isozyme A; OSHS, *O*-succinyl-L-homoserine; 5-MTHF, *5*-methyltetrahydrofolate; SAM, *S*-adenosylmethionine; SAH, *S*-Adenosylhomocysteine; SAHH, *S*-adenosylhomocysteine hydrolase.

Sulfur-containing amino acids (SCAAs), primarily cysteine and methionine, play indispensable roles in cellular physiology [1]. They are not only fundamental building blocks for protein synthesis but also serve as central players in redox homeostasis, one-carbon metabolism, and the synthesis of numerous biomolecules [1,17]. As a model organism in microbiological research, *E. coli* provides a classic paradigm for understanding the synthesis and regulatory networks of these critical amino acids [18]. This review will delve into the biosynthetic pathways of cysteine and methionine in *E. coli*, focusing on the key enzymatic steps, intricate regulatory mechanisms, and the tight interplay between these two metabolic routes (Figure 1).

### 2.1. De Novo Biosynthesis of Cysteine: A Tightly Regulated Two-Step Pathway

In *E. coli*, the de novo biosynthesis of cysteine is an efficient and tightly regulated two-step process that converts serine, a product of central carbon metabolism, into the sulfur-containing amino acid cysteine, thereby integrating inorganic sulfur into biomolecules [19].

#### 2.1.1. Initial Step: Acetylation Catalyzed by Serine Acetyltransferase (CysE)

Cysteine biosynthesis begins with the activation of L-serine, a reaction catalyzed by Serine *O*-acetyltransferase (SAT), the product of the *cysE* gene [19]. This enzyme utilizes acetyl-coenzyme A (acetyl-CoA) as the acetyl group donor to acetylate the hydroxyl group of L-serine, yielding the intermediate *O*-acetylserine (OAS) [20]. This reaction serves as the committed and primary regulatory point of the entire pathway, as its activity is subject to potent allosteric feedback inhibition by the final product, L-cysteine [21,22]. This inhibition involves a dual mechanism: cysteine not only acts as a competitive inhibitor with respect to serine by binding to the active site but also induces a conformational change in the enzyme’s C-terminal tail, which physically blocks the binding of acetyl-CoA [23]. This ensures a rapid and complete shutdown of the pathway when cysteine is abundant [21,23]. Such stringent regulation is crucial to prevent the excessive accumulation of intracellular cysteine, as high concentrations can fuel the Fenton reaction, producing cytotoxic hydroxyl radicals that cause oxidative damage to the cell [24].

#### 2.1.2. Final Step: Sulfhydration Catalyzed by Cysteine Synthase (CysK)

The second and final step of the biosynthetic pathway involves the integration of a sulfur atom into the carbon backbone [22]. This reaction is catalyzed by *O*-acetylserine sulfhydrylase, specifically the isozyme A (OASS-A) encoded by the *cysK* gene [21]. CysK is a pyridoxal 5′-phosphate (PLP)-dependent enzyme that catalyzes a β-replacement reaction, substituting the acetoxy group of OAS with sulfide (H_2_S) to ultimately produce L-cysteine and acetate [23].

#### 2.1.3. The Cysteine Synthase Complex (CSC): A Central Regulatory Hub for Sulfur Metabolism

In addition to their independent catalytic functions, CysE and CysK can form a multi-enzyme complex through high-affinity protein–protein interactions, known as the Cysteine Synthase Complex (CSC) [25]. In *E. coli*, the formation of the CSC is mediated by the insertion of the flexible C-terminal tail of CysE into the active site of CysK [26]. This interaction has profound regulatory effects, primarily activating CysE by relieving its feedback inhibition by L-cysteine and substrate inhibition by L-serine. This ensures a continuous supply of the intermediate OAS. While the complex formation may modulate CysK activity, its primary role in bacteria is often associated with substrate channeling and metabolic efficiency. This sophisticated regulation allows the cell to fine-tune cysteine biosynthesis. When sulfur is scarce, OAS accumulates, acting as a signaling molecule to activate the transcriptional regulator CysB, which upregulates genes for sulfate uptake and reduction [19].

### 2.2. Biosynthesis of Methionine: A Convergent Pathway Integrating Carbon, Sulfur, and Methyl Sources

In contrast to the metabolic pathway in mammals, the sulfur atom of methionine in *E. coli* is derived from cysteine, representing a reversal of the flow observed in mammals where methionine serves as the sulfur donor for cysteine [27]. The biosynthesis of methionine is a pathway that converges distinct metabolic precursors, with its carbon skeleton, sulfur atom, and methyl group originating from aspartate, cysteine, and the one-carbon pool, respectively [28].

#### 2.2.1. Source of the Carbon Skeleton: The Aspartate Pathway

The carbon skeleton of methionine is derived from aspartate through a series of reactions in the aspartate family amino acid biosynthetic pathway [29]. In *E. coli*, the first and third steps in the conversion of aspartate to the key intermediate homoserine are catalyzed by a unique multifunctional enzyme, aspartokinase I/homoserine dehydrogenase I, encoded by the *thrA* gene [30]. This bifunctional enzyme serves as a critical metabolic control point, its activity is allosterically feedback-inhibited by L-threonine, an end product of the aspartate pathway, thereby precisely controlling the carbon flux into this metabolic network [31]. Homoserine sits at a metabolic branch point and can be converted to either threonine or methionine [32]. The first committed step into the methionine-specific branch is the succinylation of homoserine, catalyzed by homoserine *O*-succinyltransferase (encoded by the *metA* gene), to produce *O*-succinyl-L-homoserine (OSHS) [33]. This step activates the γ-carbon of homoserine, preparing it for the subsequent transfer of the sulfur atom.

#### 2.2.2. Transsulfuration: Transfer of the Sulfur Atom from Cysteine to Homocysteine

The transfer of the sulfur atom from cysteine to the methionine precursor is accomplished through a process known as transsulfuration [34]. This pathway is catalyzed by two sequential PLP-dependent enzymes [35]. First, cystathionine γ-synthase, encoded by the *metB* gene, catalyzes a γ-replacement reaction between OSHS and L-cysteine to form the thioether intermediate, cystathionine [34]. Subsequently, cystathionine β-lyase, encoded by the *metC* gene, cleaves cystathionine via an α, β-elimination reaction, releasing homocysteine, the direct precursor of methionine, along with pyruvate and ammonia [36].

#### 2.2.3. Final Methylation: Homocysteine to Methionine

The final step in methionine biosynthesis is the transfer of a methyl group to the thiol group of homocysteine [37]. This methyl group is derived from 5-MTHF [38]. To adapt to varying environmental conditions, *E. coli* has evolved two functionally distinct but mechanistically related methionine synthases [39]. In the absence of cobalamin (vitamin B_12_), the cell relies on the cobalamin-independent methionine synthase, encoded by the *metE* gene [40]. This enzyme transfers the methyl group directly from 5-MTHF to homocysteine, ensuring survival in B12-deficient environments, albeit with relatively low catalytic efficiency [41]. Conversely, when vitamin B_12_ is available, *E. coli* preferentially utilizes the cobalamin-dependent methionine synthase, encoded by the *metH* gene [39]. MetH exhibits a catalytic efficiency approximately 40- to 100-fold higher than that of MetE. By utilizing the cobalamin cofactor as an intermediate methyl carrier, MetH significantly accelerates methionine synthesis, providing a substantial growth advantage in nutrient-rich environments [42]. Recent studies have further shown that *E. coli* can adapt via evolutionary mutations to enhance the uptake of suboptimal cobalamin analogs, thereby maximizing the high catalytic potential of MetH [43].

### 2.3. The Activated Methyl Cycle: Methionine Regeneration and Metabolic Regulation

In *E. coli*, newly synthesized methionine serves not only as a building block for proteins but also as the entry point into a central metabolic loop known as the activated methyl cycle [44]. This cycle is responsible for generating the universal methyl donor for all cellular methylation reactions and maintaining methylation homeostasis [37].

#### 2.3.1. Synthesis and Function of S-Adenosylmethionine (SAM)

One of the primary metabolic functions of methionine is to serve as the precursor for the synthesis of SAM [45]. The synthesis of SAM is catalyzed by *S*-adenosylmethionine synthetase (MAT), encoded by the *metK* gene, in a reaction that consumes one molecule of methionine and one molecule of ATP [46]. As the principal methyl donor in the cell, SAM is involved in the methylation of various biological macromolecules, including DNA, RNA, proteins, and lipids [47]. It plays an essential role in key life activities such as the epigenetic regulation of gene expression, ribosome biogenesis, and the synthesis of secondary metabolites [48].

#### 2.3.2. Regulatory Role of the Methylation Byproduct S-Adenosylhomocysteine (SAH)

Upon donating its methyl group, SAM is converted into the byproduct SAH. Far from being inert, SAH acts as a potent competitive inhibitor of most SAM-dependent methyltransferases [49]. The accumulation of SAH competes with SAM for enzyme active sites, thereby significantly impairing cellular methylation capacity [50]. Consequently, the intracellular SAM/SAH ratio is widely regarded as a critical indicator of the cellular methylation potential.

#### 2.3.3. Closing the Cycle: Regeneration of Homocysteine

To maintain the cell’s methylation capacity and recycle the sulfur-containing backbone, SAH must be rapidly removed [51]. This critical step is catalyzed by *S*-adenosylhomocysteine hydrolase (SAHH), encoded by the *ahcY* gene [52], which mediates the reversible hydrolysis of SAH into adenosine and homocysteine. The resulting homocysteine reenters the methionine synthesis pathway, where it is re-methylated into methionine by either MetE or MetH, thereby closing the activated methyl cycle. This hydrolysis is crucial for preventing the accumulation of inhibitory SAH and serves as a central element in regulating cellular methylation homeostasis [53]. Furthermore, this cycle exemplifies the principle of metabolic economy, by regenerating homocysteine, it maximizes the utilization of sulfur-containing metabolites and avoids the significant energy expenditure required for the de novo synthesis of methionine [54]. The central importance of this cycle also renders it a vulnerable metabolic hub [55]. For instance, the T3 bacteriophage has evolved a strategy to directly inhibit the host’s MetK enzyme, thereby dismantling the entire methylation system, a finding that highlights the cycle’s critical role in host-pathogen interactions [56].

## 3. The Synthesis of Cysteine and Methionine in Yeast

Yeasts, particularly *Schizosaccharomyces pombe* and *Saccharomyces cerevisiae*, are considered powerful model organisms for studying conserved cellular processes in eukaryotes. These two evolutionarily distant species provide a unique perspective for the comparative analysis of fundamental metabolic pathways, such as the biosynthesis of sulfur-containing amino acids [57]. This article will focus on haploid strains of *Schizosaccharomyces pombe* and both haploid and diploid states of *Saccharomyces cerevisiae* to illustrate their distinct metabolic strategies for synthesizing cysteine and methionine [58]. Although both utilize aspartate as a common precursor, they exhibit significant differences in the core mechanisms for sulfur incorporation and interconversion between these critical amino acids, reflecting their respective unique evolutionary trajectories.

### 3.1. De Novo Synthesis Pathway in Schizosaccharomyces pombe

**Figure 2:**
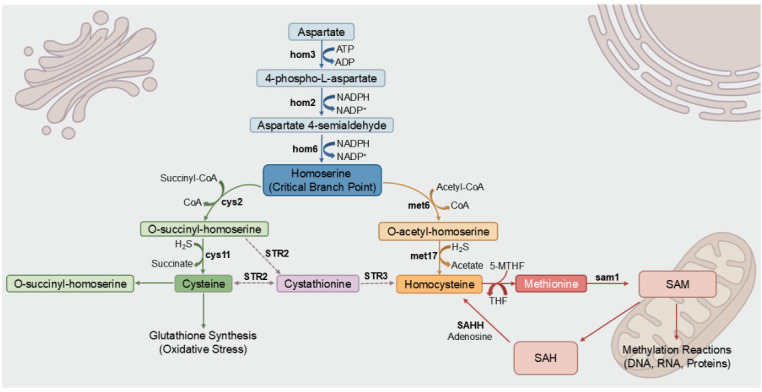
The synthesis of cysteine and methionine in *Schizosaccharomyces pombe*. Abbreviations: Acetyl-CoA, Acetyl-coenzyme A; 5-MTHF, *5*-methyltetrahydrofolate; THF, Tetrahydrofolate; SAM, *S*-adenosylmethionine; SAH, *S*-Adenosylhomocysteine; SAHH, *S*-adenosylhomocysteine hydrolase.

In *Schizosaccharomyces pombe*, the biosynthesis of cysteine and methionine originates from aspartate and inorganic sulfur, assimilated as H_2_S, following a pathway characterized by linear and parallel branches (Figure 2). The upstream portion of this pathway represents a conserved metabolic module where aspartate is converted to homoserine through three enzymatic steps. This sequence generates a critical branch-point intermediate for the biosynthesis of both sulfur-containing amino acids and threonine [59]. The subpathway is initiated by aspartate kinase, encoded by the *hom3* gene, which utilizes ATP to phosphorylate aspartate into *4*-phospho-L-aspartate [60]. Subsequently, aspartate-semialdehyde dehydrogenase, which is encoded by hom2, reduces *4*-phospho-L-aspartate to aspartate *4*-semialdehyde, using NAD(P)H as a cofactor [59]. Finally, homoserine dehydrogenase, which is encoded by hom6, catalyzes the reduction of aspartate *4*-semialdehyde to yield homoserine.

The pathway bifurcates from homoserine into two parallel branches dedicated to the de novo synthesis of cysteine and homocysteine [57]. In the cysteine branch, Cys2-mediated succinylation converts homoserine into *O*-succinyl-homoserine using succinyl-CoA. Subsequently, Cys11 catalyzes the condensation of *O*-succinyl-homoserine with hydrogen sulfide to yield cysteine and succinate [61]. Analogously, the methionine branch initiates with the acetylation of homoserine by Met6. The resulting *O*-acetyl-homoserine is then converted into homocysteine and acetate by Met17 in the presence of hydrogen sulfide [62]. Such a parallel architecture enables the independent regulation of metabolic flux, allowing cells to balance demands for cysteine and methionine [57].

In addition to de novo synthesis from *O*-acetyl-homoserine, homocysteine can be derived from cystathionine via an alternative route. This pathway initiates with *O*-succinyl-homoserine and cysteine, mediated by the STR2 and STR3 enzymes [63]. Once synthesized, homocysteine enters the final stage of methionine biosynthesis. This step is catalyzed by *5*-methyltetrahydropteroyltriglutamate-homocysteine methyltransferase, encoded by the *met26* gene, which transfers a methyl group from 5-MTHF to homocysteine to form methionine [64].

Methionine subsequently plays a pivotal role in the methionine cycle, where it is converted into SAM by *S*-adenosylmethionine synthase, encoded by the *sam1* gene [65]. As the primary methyl donor in the cell, SAM participates in the methylation of diverse biomolecules, including DNA, RNA, and proteins. This process is critical for the regulation of gene expression and cell signaling [66]. Following methyl transfer, SAM is converted to SAH. SAH is subsequently hydrolyzed by SAHH into homocysteine and adenosine, thereby completing the cycle and regenerating the precursor for methionine synthesis [51].

### 3.2. Homocysteine-Centric Metabolic Hub in Saccharomyces cerevisiae

**Figure 3:**
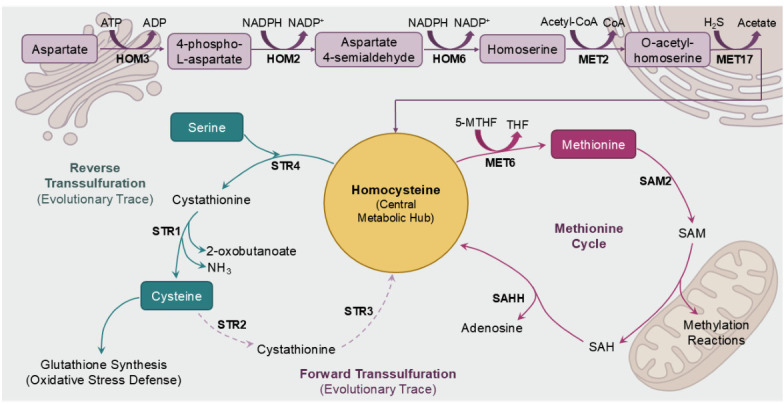
The synthesis of cysteine and methionine in *Saccharomyces cerevisiae*. Abbreviations: Acetyl-CoA, Acetyl-coenzyme A; 5-MTHF, *5*-methyltetrahydrofolate; THF, Tetrahydrofolate; SAM, *S*-adenosylmethionine; SAH, *S*-Adenosylhomocysteine; SAHH, *S*-adenosylhomocysteine hydrolase.

In contrast to *S. pombe*, the pathway for homocysteine synthesis from aspartate is the key upstream route for the synthesis of both cysteine and methionine in *Saccharomyces cerevisiae*, making homocysteine a central metabolic hub (Figure 3). Here, the sulfur atom of homocysteine is transferred for cysteine synthesis, while its carbon skeleton is methylated to form methionine [67]. Cysteine synthesis in *S. cerevisiae* relies exclusively on the so-called reverse transsulfuration pathway [68]. The first step of this pathway is catalyzed by cystathionine β-synthase, encoded by the *STR4* gene, which condenses serine and homocysteine to form cystathionine [69]. Subsequently, cystathionine γ-lyase, encoded by the *STR1* gene, catalyzes the cleavage of cystathionine to produce cysteine, *2*-oxobutanoate, and ammonia [70]. This pathway is critical for maintaining cellular cysteine levels, as cysteine serves as a precursor for glutathione synthesis, which plays a central role in combating oxidative stress [71].

Concurrently, another portion of homocysteine is utilized for methionine synthesis. This process is catalyzed by *5*-methyltetrahydropteroyltriglutamate-homocysteine methyltransferase, which is encoded by the *MET6* gene. The enzyme transfers a methyl group from 5-MTHF [72]. Metabolic engineering of MET6 and SAM2 can significantly enhance intracellular SAM production, highlighting the importance of this step [73]. Furthermore, *Saccharomyces cerevisiae* retains the ability to synthesize homocysteine from cysteine via cystathionine γ-synthase and cystathionine β-lyase [74]. In contrast to the cyclic methionine cycle, the conversion between homocysteine and cysteine mediated by STR1, STR2, STR3 and STR4 is not a cyclic process [75]. Instead, the forward transsulfuration pathway catalyzed by STR2 and STR3 does not serve as a primary anabolic route in *S. cerevisiae*, it resembles an evolutionary remnant, possibly reflecting the organism’s ancestral reliance on this pathway for sulfur metabolism [76].

## 4. The Synthesis of Cysteine and Methionine in *Arabidopsis thaliana*

**Figure 4:**
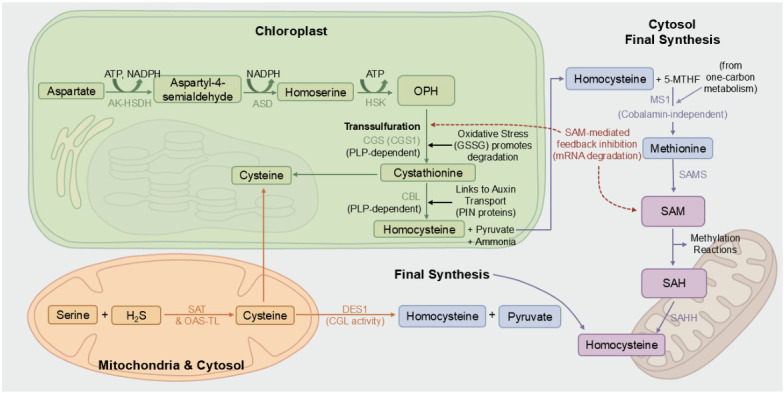
The synthesis of cysteine and methionine in *Arabidopsis thaliana*. Abbreviations: AK-HSDH, Aspartate kinase-homoserine dehydrogenase; ASD, Aspartate semialdehyde dehydrogenase; HSK, Homoserine kinase; OPH, *O*-phosphohomoserine; CGS, Cystathionine γ-synthase; GSSG, Oxidized glutathione; CBL, Cystathionine β-lyase; H_2_S, sulfide; SAT, Serine *O*-acetyltransferase; OAS-TL, *O*-acetylserine (thiol) lyase; DES1, Desulfhydrase 1; CGL, Cystathionine γ-lyase; 5-MTHF, *5*-methyltetrahydrofolate; MS1, Methyltransferase 1; SAMS, *S*-Adenosylmethionine Synthase; SAM, *S*-adenosylmethionine; SAH, *S*-Adenosylhomocysteine; SAHH, *S*-adenosylhomocysteine hydrolase.

In plants, the de novo synthesis of methionine is fundamentally distinct from the pathway in mammals, as it relies on cysteine to serve as the essential sulfur donor through a process known as the transsulfuration pathway [77]. This metabolic route represents a critical intersection of carbon, nitrogen, and sulfur assimilation, requiring coordinated input from multiple primary metabolic pathways (Figure 4). The two key substrates, *O*-phosphohomoserine (OPH) and cysteine, are derived from separate biosynthetic origins [78,79]. OPH is synthesized from aspartate within the chloroplast through a series of reactions catalyzed by the bifunctional aspartate kinase-homoserine dehydrogenase (AK-HSDH), aspartate semialdehyde dehydrogenase (ASD), and homoserine kinase (HSK) [80]. Concurrently, cysteine is produced in the cytosol, mitochondria, and plastids from serine and inorganic sulfide via the sequential action of SAT and *O*-acetylserine (thiol) lyase (OAS-TL) [81]. This compartmentalization, with early steps occurring in the chloroplast and the final methylation step in the cytosol, necessitates inter-organellar transport of intermediates and allows for complex, multilayered regulation that links photosynthetic activity directly to the production of this essential amino acid [82].

The first committed and primary regulatory step in the methionine-specific branch is the formation of the thioether intermediate, cystathionine, from OPH and cysteine [83]. This γ-replacement reaction is catalyzed by the chloroplastic enzyme cystathionine γ-synthase (CGS), which is encoded by the *CGS1* gene. As a PLP-dependent enzyme, Arabidopsis CGS is subject to sophisticated regulation that integrates signals related to both metabolic homeostasis and cellular stress [84]. The principal control mechanism is a post-transcriptional feedback loop mediated by SAM, the main downstream product of methionine metabolism [85]. An excess of SAM induces translational arrest of the CGS1 mRNA, which subsequently triggers its degradation, thereby rapidly downregulating enzyme synthesis to match cellular demand for methylation reactions [86]. Furthermore, CGS activity is modulated by the cellular redox state. Under oxidative stress, elevated levels of oxidized glutathione (GSSG) can bind to and accelerate the degradation of the CGS protein, effectively rerouting the limited cysteine pool away from methionine synthesis and towards the more immediate need for glutathione production for antioxidant defense [87].

Following its synthesis in the chloroplast, cystathionine is cleaved in the penultimate step of the pathway to yield homocysteine, pyruvate, and ammonia. This α, β-elimination reaction is catalyzed by another plastid-localized, PLP-dependent enzyme, cystathionine β-lyase (CBL) [88]. The essentiality of CBL is highlighted by studies showing that its inhibition or genetic knockout leads to methionine starvation, resulting in either a lethal phenotype or severely stunted plant growth. The catalytic mechanism of CBL relies on the PLP cofactor to facilitate the Cβ-Sγ bond cleavage of cystathionine through the formation of a stabilized quinoid intermediate [89]. Beyond its canonical role in methionine biosynthesis, recent evidence has linked CBL function to broader developmental processes. For instance, disruption of CBL activity in Arabidopsis has been shown to reduce the abundance of PIN-FORMED (PIN) proteins, which are critical transporters for the phytohormone auxin, thereby impairing auxin-responsive gene expression and connecting sulfur metabolism directly to hormonal regulation of plant architecture [90].

The final step of de novo methionine synthesis, the methylation of homocysteine, occurs in the cytosol, requiring the export of homocysteine from the chloroplast [91]. This reaction is catalyzed by the enzyme *5*-methyltetrahydropteroyltriglutamate-homocysteine methyltransferase 1 (MS1), a cobalamin-independent methionine synthase. MS1 transfers a methyl group from 5-MTHF, a derivative of the folate-mediated one-carbon metabolism pathway, to the thiol group of homocysteine to form methionine [92]. This cytosolic localization is crucial, as the methionine produced is the immediate precursor for SAM, the universal methyl donor for virtually all cellular methylation reactions. The activity of MS1 is therefore critical for maintaining the cellular methylation potential, often measured as the SAM-to-SAH ratio [93]. Indeed, mutations in the *ATMS1* gene cause a significant decrease in this ratio, leading to genome-wide reductions in DNA methylation and histone H3 lysine 9 dimethylation. These epigenetic changes, in turn, affect chromatin silencing, gene expression, and key developmental processes such as seed germination [94].

While the primary metabolic flux in plants is directed towards methionine synthesis, the existence of the bifunctional enzyme L-cysteine desulfhydrase 1 (DES1) introduces a layer of metabolic plasticity. The principal and well-established role of DES1 is the cytosolic degradation of cysteine into pyruvate, ammonia, and the gaseous signaling molecule H_2_S [95]. This function is integral to cellular signaling, particularly in the abscisic acid (ABA)-dependent pathway for stomatal closure [96]. However, DES1 has also been shown to possess cystathionine γ-lyase (CGL) activity, the key enzymatic function of the reverse transsulfuration pathway from methionine to cysteine that is dominant in mammals [97]. This CGL activity suggests that Arabidopsis retains the enzymatic capacity to convert cystathionine back to cysteine. While not a primary route, this bifunctionality may allow the plant to salvage sulfur from the methionine pathway or fine-tune the balance of sulfur-containing amino acids under specific metabolic conditions, thereby providing a mechanism for homeostatic regulation at the cystathionine metabolic node [98].

## 5. The Synthesis of Cysteine and Methionine in *Homo sapiens*

**Figure 5:**
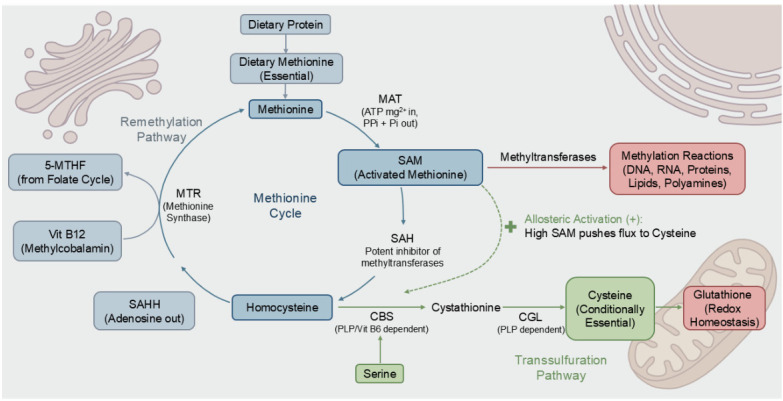
The synthesis of cysteine and methionine in *Homo sapiens*. Abbreviations: 5-MTHF, *5*-methyltetrahydrofolate; SAHH, *S*-adenosylhomocysteine hydrolase; CBS, Cystathionine β-synthase; SAH, *S*-Adenosylhomocysteine; SAM, *S*-adenosylmethionine; MAT, *S*-adenosylmethionine synthetase; CGL, Cystathionine γ-lyase.

Before diving into the specific metabolic limitations of *Homo sapiens*, it is crucial to consider the evolutionary context of this pathway in other multicellular heterotrophs, such as the nematode *Caenorhabditis elegans* and the fruit fly *Drosophila melanogaster*. Unlike autotrophic plants, these invertebrate models, much like mammals, heavily rely on dietary sources of methionine as the primary precursor to drive the energetically costly biosynthesis of cysteine. In these organisms, the transsulfuration pathway is highly conserved, representing a fundamental evolutionary strategy to manage the intense energetic costs associated with sulfur amino acid interconversion before the emergence of highly specialized mammalian networks. Building upon this conserved foundation, in *H. sapiens*, the metabolism of sulfur-containing amino acids differs significantly from that of other organisms due to these specific evolutionary and biochemical constraints. To elucidate this complex process, the figure below summarizes the key enzymes involved in methionine and cysteine metabolism and their properties (Figure 5).

### 5.1. Metabolic Limitations and the Essentiality of Methionine in Humans

Unlike *E. coli* and higher plants, which synthesize methionine de novo from precursors such as aspartate, mammals lack this pathway [99]. Consequently, methionine is an essential amino acid dependent on dietary intake [100], serving as the ultimate sulfur donor for all sulfur-containing amino acids in humans [101]. Conversely, cysteine is classified as a conditionally non-essential amino acid [102]. Its synthesis relies exclusively on the transsulfuration pathway, which converts methionine-derived homocysteine (Hcy) and serine into cysteine [103]. Thus, cysteine synthesis is strictly contingent upon methionine availability, and insufficient methionine intake renders cysteine essential [104]. This dependency positions dietary methionine at a critical metabolic nexus, influencing not only protein synthesis but also cellular epigenetic potential and redox homeostasis [105].

### 5.2. The Methionine Cycle: The Sole Endogenous Source of Homocysteine

Homocysteine, a non-proteinogenic sulfur-containing amino acid, serves as the central hub linking methionine and cysteine metabolism. Not derived from the diet, homocysteine is formed exclusively as an intermediate within the methionine cycle [106]. This cycle, initiated by methionine activation, positions homocysteine at a critical metabolic branch point where it faces two competing fates: remethylation back to methionine or irreversible catabolism via the transsulfuration pathway to synthesize cysteine [103]. Given that the metabolic flux of both essential methionine and conditionally essential cysteine converges at this node, the precise regulation of homocysteine is vital for cellular homeostasis. Consequently, plasma homocysteine concentration acts as a sensitive biomarker of one-carbon metabolic status, and elevated levels typically reflect enzymatic disruptions or vitamin deficiencies rather than direct dietary intake [107].

### 5.3. S-Adenosylmethionine (SAM): Synthesis and Its Role as a Universal Methyl Donor

Beyond its role as a fundamental building block for proteins, the majority of methionine in the human body is utilized for the synthesis of SAM, a high-energy compound often referred to as activated methionine [108]. The biosynthesis of SAM is an ATP-dependent process catalyzed by MAT, which is encoded by the *MAT1A* and *MAT2A* genes in humans [109]. This enzymatic reaction is unique in that it transfers the entire adenosyl group from an ATP molecule to the sulfur atom of methionine, followed by the hydrolysis of the resulting triphosphate, making the reaction virtually irreversible under physiological conditions [110]. The most critical function of SAM is to serve as the universal methyl donor for the vast majority of methylation reactions within the cell [9]. These reactions, catalyzed by methyltransferases, are fundamental to cellular regulation, encompassing a wide range of vital activities from the epigenetic modification of DNA and histones to the synthesis of neurotransmitters [111]. Furthermore, SAM also serves as a donor of aminopropyl groups for the synthesis of polyamines, which are essential molecules for cell growth and proliferation [112].

### 5.4. The Path to Homocysteine: SAH Formation and Hydrolysis

Following its role as a methyl donor, SAM is converted into SAH, a potent feedback inhibitor of most methyltransferases [113]. The accumulation of SAH would halt cellular methylation reactions. Therefore, its efficient removal is crucial for maintaining methylation capacity [114]. This task is executed by SAHH, which catalyzes the reversible hydrolysis of SAH into adenosine and homocysteine [115]. Although the equilibrium of this reaction theoretically favors SAH synthesis, in vivo it proceeds toward hydrolysis because the products adenosine and homocysteine are rapidly scavenged by downstream pathways. This step completes the methionine cycle, releasing homocysteine back into the metabolic pool for further metabolism. Consequently, the intracellular SAM: SAH ratio often referred to as the methylation index, is considered a more accurate indicator of the cell’s methylation capacity than SAM levels alone [116].

### 5.5. Regulation at the Crossroads: The Metabolic Fate of Homocysteine

Homocysteine occupies a critical metabolic branch point, where it can be directed into two distinct pathways [117]. The first is remethylation to methionine, a process that conserves both the sulfur atom and the carbon skeleton [118]. This reaction is primarily catalyzed by methionine synthase (MS), which utilizes methylcobalamin as a coenzyme and 5-MTHF as the methyl donor, thereby coupling the methionine cycle with folate metabolism [119]. The second pathway is the irreversible transsulfuration pathway [120]. The initial step, catalyzed by cystathionine β-synthase (CBS), involves the condensation of homocysteine with serine to form cystathionine, requiring PLP as a cofactor [121].

The metabolic fate of homocysteine is predominantly regulated by SAM [122,123]. Elevated intracellular SAM levels, indicative of methionine abundance, act as an allosteric activator of CBS [124]. This feed-forward mechanism ensures that excess homocysteine is diverted into the transsulfuration pathway for the synthesis of cysteine, glutathione, and other sulfur-containing compounds [125]. Conversely, low SAM levels reduce CBS activity, thereby channeling the limited homocysteine pool toward remethylation to regenerate the essential amino acid methionine [126].

## 6. Conclusions and Perspectives

Throughout cysteine and methionine metabolism, H_2_S serves as the primary source of sulfur [127]. A key distinction lies in their functional groups: the sulfur atom in cysteine enables the regulation of redox balance, whereas the sulfur atom in methionine facilitates its role as a methyl carrier [128]. In the methionine cycle, methionine synthase catalyzes the synthesis of methionine from homocysteine. Subsequently, methionine-derived SAM donates a methyl group in methylation reactions and is converted to SAH. SAH is then hydrolyzed to regenerate homocysteine [129]. Notably, in organisms capable of de novo methionine synthesis, this pathway is often referred to as the homocysteine cycle, as homocysteine marks the starting point [130].

Beyond methylation, the homocysteine cycle demonstrates metabolic economy. In mammals, cysteine is classified as a non-essential amino acid because it can be endogenously synthesized from methionine-derived homocysteine and serine via the transsulfuration pathway, catalyzed by CBS and cystathionine γ-lyase (CTH) [15]. Consequently, mammalian cysteine synthesis differs from that in other organisms [131]. Although the primary function of the transsulfuration pathway is cysteine biosynthesis, it has been discovered that this pathway also contributes to endogenous H_2_S production through the enzymatic activities of CBS and CTH [132]. This suggests a potential regulatory role of the transsulfuration pathway in methionine metabolism [133]. From a broader perspective, dietary methionine consumed by mammals originates from plant synthesis. The sulfur, initially assimilated as H_2_S or cysteine in plants, is released back into the metabolic pool as H_2_S and cysteine in mammals via the actions of CBS and CTH [134].

From a biosynthetic perspective, mammals, including humans have lost the capacity for de novo methionine synthesis, rendering it an essential amino acid. The ancestral pathway of methionine biosynthesis from aspartate is energetically costly and inefficient, making dietary acquisition a more economical strategy given the abundance of food sources [135,136]. Consequently, several amino acids, including methionine, have become essential due to the loss of their synthetic pathways [137]. In contrast, the de novo synthesis of cysteine presents a paradox [138]. While the direct synthesis of cysteine from serine and H_2_S is theoretically straightforward, mammals primarily rely on the transsulfuration pathway, which uses methionine as a sulfur donor. This pathway is metabolically costly and inefficient [139,140]. Notably, in non-small cell lung cancer (NSCLC) cell lines, serine fails to contribute to cysteine synthesis via the transsulfuration pathway [141]. Furthermore, the conversion of methionine to cysteine is limited by SAM-mediated methylation regulation. Intriguingly, the two key enzymes in this pathway, CBS and CTH, exhibit higher activity in catalyzing H_2_S production from cysteine than in generating cysteine [142].

In summary, while metabolic evolution generally favors efficiency and economy, the retention of an energetically costly cysteine biosynthetic pathway in mammals suggests a compromise, possibly for the sake of regulatory control or H_2_S signaling. While the underlying evolutionary rationale remains an open question, elucidating these complex metabolic networks extends far beyond fundamental physiology. A comprehensive understanding of sulfur amino acid conversion is crucially important for industrial biotechnology and pharmaceutical manufacturing. Specifically, mapping these metabolic pathways is essential for optimizing the precursor supply required for the biosynthesis of antibiotics and other active compounds. As highlighted in recent studies on industrial microorganisms like Streptomyces, strategically manipulating sulfur metabolism significantly enhances the yield of valuable secondary metabolites [143]. Ultimately, translating this metabolic knowledge into targeted metabolic engineering offers highly promising avenues for advanced drug production.

## Data Availability

No new data were created or analyzed in this study. Data sharing is not applicable to this article.

## Abbreviations

The following abbreviations are used in this manuscript:

GSH	Glutathione
SAM	S-adenosylmethionine
SCAAs	Sulfur-containing amino acids
SAT	Serine O-acetyltransferase
Acetyl-CoA	Acetyl-coenzyme A
OAS	O-acetylserine
PLP	Pyridoxal 5′-phosphate
H_2_S	Sulfide
CSC	Cysteine Synthase Complex
OSHS	O-succinyl-L-homoserine
5-MTHF	5-methyltetrahydrofolate
MAT	S-adenosylmethionine synthetase
SAH	S-Adenosylhomocysteine
SAHH	S-adenosylhomocysteine hydrolase
OPH	O-phosphohomoserine
AK-HSDH	Aspartate kinase–homoserine dehydrogenase
ASD	Aspartate semialdehyde dehydrogenase
HSK	Homoserine kinase
OAS-TL	O-acetylserine (thiol) lyase
CGS	Cystathionine γ-synthase
GSSG	Oxidized glutathione
CBL	Cystathionine β-lyase
PIN	PIN-FORMED
MS1	Methyltransferase 1
DES1	Desulfhydrase 1
ABA	Abscisic acid
CGL	Cystathionine γ-lyase
Hcy	Homocysteine
MS	Methionine synthase
CBS	Cystathionine β-synthase
CTH	Cystathionine γ-lyase
NSCLC	Non-small cell lung cancer
